# Auditory Deficits in Audiovisual Speech Perception in Adult Asperger’s Syndrome: fMRI Study

**DOI:** 10.3389/fpsyg.2019.02286

**Published:** 2019-10-10

**Authors:** Fabian-Alexander Tietze, Laura Hundertmark, Mandy Roy, Michael Zerr, Christopher Sinke, Daniel Wiswede, Martin Walter, Thomas F. Münte, Gregor R. Szycik

**Affiliations:** ^1^Department of Psychiatry, Social Psychiatry and Psychotherapy, Hannover Medical School, Hanover, Germany; ^2^Asklepios Clinic North – Ochsenzoll, Hamburg, Germany; ^3^Department of Psychosomatic Medicine and Psychotherapy, Hannover Medical School, Hanover, Germany; ^4^Institute of Psychology II, University of Lübeck, Lübeck, Germany; ^5^Department of Neurology, University of Lübeck, Lübeck, Germany; ^6^Department of Psychiatry and Psychotherapy, University of Tübingen, Tübingen, Germany

**Keywords:** Asperger’s syndrome, autism, audiovisual, speech, multisensory, fMRI

## Abstract

Audiovisual (AV) integration deficits have been proposed to underlie difficulties in speech perception in Asperger’s syndrome (AS). It is not known, if the AV deficits are related to alterations in sensory processing at the level of unisensory processing or at levels of conjoint multisensory processing. Functional Magnetic-resonance images (MRI) was performed in 16 adult subjects with AS and 16 healthy controls (HC) matched for age, gender, and verbal IQ as they were exposed to disyllabic AV congruent and AV incongruent nouns. A simple semantic categorization task was used to ensure subjects’ attention to the stimuli. The left auditory cortex (BA41) showed stronger activation in HC than in subjects with AS with no interaction regarding AV congruency. This suggests that alterations in auditory processing in unimodal low-level areas underlie AV speech perception deficits in AS. Whether this is signaling a difficulty in the deployment of attention remains to be demonstrated.

## Introduction

Speech perception under natural conditions relies on auditory and on visual information. In particular, speech lip movements impact speech perception as powerfully demonstrated by the so called McGurk-illusion (McGurk and MacDonald, 1976). But audiovisual (AV) perception does not only play a role in artificial illusions. Under noisy conditions AV information (in comparison to auditory only information) results in considerable improvements in intelligibility (Sumby and Pollack, 1954; Schwartz et al., 2004; Ross et al., 2007a). In complex auditory environments, such as the proverbial cocktail-party, visual cues may enhance selective listening (Driver, 1996). Combining multisensory information is crucial not only for perception of speech but also for perception of other environmental events (e.g., traffic noise in the city) and provides different evolutionary benefits, like rapid stimulus detection (Gondan et al., 2005; Diederich and Colonius, 2015) and localization (Hairston et al., 2003). Thus intact AV processing is crucial for swift interaction with the environment and also serves important social functions.

Deficits in AV perception have been shown for diverse populations with neuropsychiatric disorders. Individuals with dyslexia show multisensory processing deficits with reduced ability to integrate AV information (Hahn et al., 2014; Rüsseler et al., 2015, 2018; Ye et al., 2017). Likewise, schizophrenia has also been reported to lead to impaired AV speech perception (De Gelder et al., 2003; Ross et al., 2007b; Szycik et al., 2009b, 2013) and to prolonged reaction times in AV target detection tasks (Williams et al., 2010). Subjects with different kinds of synesthesia show reduced AV integration of simple AV stimuli (Neufeld et al., 2012) as well as of AV speech (Sinke et al., 2014).

Autism spectrum disorder (ASD) is a neurodevelopmental condition characterized by persistent deficits in social communication and restricted, repetitive patterns of behavior, interests or activities (American Psychiatric Association, 2013). Eye contact and eye gaze is generally atypical in autism, a feature that persists into adulthood (Baron-Cohen et al., 2001a; Klin et al., 2002; Pelphrey et al., 2005; Pierce et al., 2011; Jones and Klin, 2013). In addition to the impact of an impairment of gaze behavior on social contexts, these impairments might also impact AV integration of speech. Indeed, there is growing evidence for AV integration deficits in ASD (Feldman et al., 2018a). Changes in multisensory function have been demonstrated using simple auditory and visual stimuli (Brandwein et al., 2013) and the double-flash illusion (Foss-Feig et al., 2010). Subjects with ASD are less susceptible to the speech related McGurk illusion (Taylor et al., 2010; Bebko et al., 2014; Stevenson et al., 2014) and show lower sensitivity for temporal synchrony of AV speech events (Bebko et al., 2006). But the picture is not fully consistent (Zhang et al., 2018). Studies exist showing no differences in AV speech perception between ASD and typically developed subjects (Williams et al., 2004). The inconsistencies of the findings are likely a result of the heterogeneity of the studied subjects who differed often in age and severity of symptomatology of ASD across studies (Feldman et al., 2018a).

In the DSM-5 (American Psychiatric Association, 2013), the categorization into autistic disorder and Asperger’s syndrome (AS) no longer exists. Autism is now defined as ASD with three gradient levels of severity categorized according to the severity of symptoms and need of support. Until the publication of DSM-5 in 2013, the former DSM-IV-TR subsumed at least two different main-subcategories of ASD within pervasive development disorders, i.e., autistic disorder and AS. Thereby diagnosis of autistic disorder was related to a delay in the development of spoken language. According to the DSM-5, individuals with an assured DSM-IV-TR diagnosis of an AS shall now be classified as ASD. Therefore results of earlier studies have to be interpreted keeping in mind the fact that some of them analyzed only subpopulations of the recent ASD group. In this manuscript we refer to ASD according to DSM-5 and AS as a subpopulation of ASD according to DSM-IV-TR.

Whereas children with AS showed a regular rate of McGurk illusions (Bebko et al., 2014), adolescent ASD subjects (Stevenson et al., 2014) showed reduced rates and adult AS subjects have been shown to have qualitative differences in perception of McGurk stimuli compared to control subjects (Saalasti et al., 2012) and to show further differences in AV speech perception (Saalasti et al., 2011). On the other hand children with AS also show deficits in speech perception (Saalasti et al., 2008) and these deficits seem to be amplified in acoustically noisy environments (Alcantara et al., 2004), i.e., situations in which visual information is most important (Ross et al., 2007a). Thus, autistic children have less benefit from AV information in speech perception of whole words whereas perception of phonemes is almost intact (Stevenson et al., 2017). The authors interpret their finding in the manner of affected sensory processing as origin for reduced multisensory abilities in autism. This may be reflected in atypical patterns of sensory responsiveness related to multisensory speech perception and integration problems (Feldman et al., 2018b). Interestingly, AV deficits in speech perception seem to disappear in early adolescence (Foxe et al., 2015). It is therefore possible that multisensory integration problems are directly related to disturbed or prolonged maturation of the sensory system in AS (Brandwein et al., 2015) or ASD (Beker et al., 2018). This could be dependent on the specific subtype respective severity level when taking into account the genetic heterogeneity of the ASD population (Robertson and Baron-Cohen, 2017) or specific age of analyzed subjects (Beker et al., 2018; Feldman et al., 2018a).

To summarize, the knowledge about AV integration deficits in ASD and especially AS is quite sparse and ambiguous with growing evidence for worse performance within the ASD population compared to typically developed subjects. These deficits are more pronounced in younger subjects and are correlated with autism symptom severity. Recently different mechanisms underlying these deficits have been postulated (Robertson and Baron-Cohen, 2017). These mechanisms can be subdivided in sensory-first and top-down accounts.

Within the sensory-first account AV integration deficits have been attributed to unisensory deficits, e.g., a weaker visual influence during multisensory processing, conceptualized as a rather peripheral problem (Gelder et al., 1991). This reduction affects predominantly tasks involving human faces or socially relevant stimuli (Mongillo et al., 2008; Irwin et al., 2011) and may be also based on the specific deficit related to the ability to process biological motion (Saalasti et al., 2012) or problems with processing temporal aspects of sensory information (Kwakye et al., 2011; Woynaroski et al., 2013).

Top-down accounts explain such AV integration deficits by problems with combining stimulus details or specific features into a coherent percept as it is postulated by the “weak central coherence theory” (Happe and Frith, 2006). Following the theory of weak central coherence, unisensory representations of specific environmental objects or events are not or rather not sufficiently integrated into a multisensory representation of these objects or events (Megnin et al., 2012). In this case alterations in functionality of typical multisensory brain areas could be responsible for AV integration deficits.

To identify brain areas responsible for AV speech integration in AS, we used functional magnetic resonance imaging (fMRI) in adult AS subjects who were exposed to AV congruent (video and audio signal matched, e.g., visual: hotel, auditory: hotel) and incongruent speech (video and audio signal did not match, e.g., visual: island, auditory: hotel) stimulation. As far as unisensory deficits underlie multisensory integration problems in AS, this should be reflected in activity differences within specific unisensory brain areas. Otherwise differences within typical multisensory brain areas should be detected.

## Materials and Methods

All procedures had been approved by the local Ethics Committee of the Hannover Medical School and have been performed in accordance with the ethical standards laid down in the Declaration of Helsinki. The participants gave written informed consent and participated for a small monetary compensation.

### Participants

Participants were divided into two groups: 16 adult subjects diagnosed with AS meeting the DSM-IV-TR criteria (American Psychiatric Association, 2000) and 16 age-matched healthy controls (HC). Nine of the AS subjects met DSM-IV-TR criteria for one or more additional psychiatric diagnoses (agoraphobia, *n* = 2; social phobia, *n* = 2; somatization disorder, *n* = 1; depression, *n* = 2; attention deficit hyperactivity disorder, *n* = 1; dysthymia, *n* = 2; and obsessive-compulsive disorder, *n* = 1). All HC subjects were free of previous and recent neurological or psychiatric diseases. Two additional AS subjects were excluded from the study due to excessive movements (more than 3 mm or 3° rotation) within the scanner (*n* = 1) and incomplete fulfilment of diagnostic criteria (*n* = 1). Two additional HC subjects were excluded because of excessive movement during MRI data acquisition. All AS subjects were recruited from the long-standing patient pool of the Department of Psychiatry of Hannover Medical School. All of them were consulted for a first-time diagnosis. As the diagnostic assessment was primarily made in the context of the psychiatric health care system and participants were recruited from that existing pool, only diagnostic data according to DSM-IV-TR-criteria was available. As shown in Table 1 both groups were matched for age, gender, and estimated verbal IQ as assessed by the MWT-B – “Mehrfachwahl-Wortschatz-Intelligenztest” (Lehrl et al., 1995).

**TABLE 1:** Characteristics of the experimental groups.

	**AS (*n* = 16)**	**HC (*n* = 16)**	**Significance**
Age in years (M, SD)	39.50, 11.17	33.75, 8.22	*t* = 1.76; n.s. (*p* = 0.09, *d* = 0.59)
No of males/females	13/3	15/1	χ^2^_0__.__05__;__1_ = 1.14; n.s. (*p* = 0.29, *V* = 0.19)
Verbal IQ^a^	30.28, 4.37	31.72, 4.10	*t* = −0.84; n.s. (*p* = 0.34, *d* = 0.34)
AQ^b^	40.37, 5.27		

*^*a*^IQ, raw score assessed by the MWT-B – “Mehrfachwahl-Wortschatz-Intelligenztest”. ^*b*^Autism-spectrum Quotient.*

All participants were native speakers of German. The mean autism-spectrum quotient (AQ) for the AS subjects group was 40.37 ± 5.27 (Baron-Cohen et al., 2001b) and thus in the higher range. Two AS subjects declared ambidexterity, whereas two HC subjects were exclusively left-handed.

DSM-IV-TR criteria for AS in adulthood (American Psychiatric Association, 2000) were thoroughly explored by a self-developed semi-structured interview (“Diagnostic interview: AS in adulthood”). After a general section focusing on medical anamnesis (somatic, psychiatric, and social histories, including childhood development), the interview continued with a special section involving AS that included the following items with respect to childhood and adulthood: social interaction and communication (e.g., friendships with/relationship to/interest in peers, and being a loner and suffering from loneliness); special interests (e.g., spending leisure time and interest in specific objects/topics); stereotypic behavior (e.g., rituals and reaction toward disturbances of rituals); and other characteristics (e.g., clumsiness and sensitivity toward noises/smells/tactile stimuli). The interview contained items and descriptions of all relevant criteria for the diagnosis of AS as defined in DSM-IV-TR. The result of the interview was confirmed for every AS-subject by verifying the threshold value of the AQ. Additionally, we observed eye contact, facial expression, prosody, and “mirroring” of affections and clumsiness during the interview. The interview was conducted by the same experienced psychiatrist and had a duration of approximately 90 min. At the time of diagnostic investigation, the investigator was blind to the research questions. Diagnosis was completed with information from personal interviews, gained by telephone or in written form, and observers in child- and/or adulthood (e.g., partners, friends, and parents or siblings). In some cases, school reports were incorporated. All DSM-IV-TR criteria had to be clearly fulfilled to confirm diagnosis. Moreover, retrospective data on the development of speech were assessed. An additional examination for axis-I co-morbidity was undertaken by using the German version of the Structured Clinical Interview for DSM-IV Axis I Disorders (SCID-I) (Wittchen et al., 1997). We had no exclusion-criteria based on medication.

### Stimuli and Design

The chosen paradigm to elicit brain activity for AV speech stimulation has been successfully used in healthy subjects and different neuropsychiatric populations (Szycik et al., 2009a, b; Rüsseler et al., 2017). Stimuli, taken from the German part of the CELEX-Database (Baayen et al., 1993), comprised 70 disyllabic nouns with a Mannheim frequency 1,000,000 (MannMln) of at least one (see, Supplementary Table S1 for the stimulus list in our study). MannMln frequency serves as frequency measure indicating the occurrence of a word within the 6,000,000 words of the Mannheim word corpus. The stimuli were spoken by a female native speaker of German with linguistic experience and recorded by means of a digital camera and a microphone. The videos (400^2^ pixels resolution, 6° visual angle) showed the frontal view of the whole face of the speaker and were divided into periods of 2 s duration, accompanied by audio streams in mono-mode. The stimuli were randomly divided into two sets of 35 items each. The first set contained video segments with congruent AV information: Lip movements were fitting to the word spoken by the speaker. The second set consisted out of video sequences with incongruent AV information: Lip movements did not fit to the spoken words; e.g., video: Engel/angel and audio: Hase/rabbit. Auditory and visual information in the incongruent stimuli started simultaneously to the onset of vocalization. The participants were instructed to carefully watch and listen to the stimuli without being informed about the AV incongruence of some stimuli. The subjects were asked to keep attention on both modalities. To ensure the attention of the subjects to the stimuli, we used a simple semantic categorization task and analyzed the detection rate (answer rate for each stimulus). Subjects were asked to respond for each stimulus by pressing the left or right response device for stimuli describing living objects (five target stimuli) vs. objects of other categories (remaining 30 stimuli). The loudness of the presented stimuli was individually adjusted to the almost audible threshold for auditory comprehension. Firstly, the interaural loudness difference (due to individually applied ear plugs) for each subject was corrected by presenting a simple test tone and changing the sound pressure level (SPL) bilaterally until subjects signaled that the audible signal was equally loud in both ears. In the second step, we presented some test stimuli during the real scanner noise and increased SPL until subjects gave us a signal by the response device that they heard the stimuli well.

Presentation software (Neurobehavioral Systems, Inc., Albany, CA, United States) was used to deliver the stimuli. A “slow event related” design was used for the stimulus presentation. Each stimulation event was followed by a fixed resting period of 16 s duration. During this time a dark screen with a fixation cross at the position of the speaker’s mouth was presented. The duration of the whole functional stimulation part of the experiment was therefore 21 min.

Presenting the stimuli and communicating between the examination and control rooms was possible due to an fMRI compatible audio system integrated into earmuffs for reduction of residual background scanner noise. Visual stimuli were presented on a MRI compatible screen positioned in the front of the scanner. Subjects were able to see the screen through a mirror positioned on the top of the head coil. To ensure good visibility a detailed test picture with similar size and resolution as the video sequences was presented prior to the experiment and all participants were asked to report the content of this picture.

### Image Acquisition

Magnetic-resonance images were acquired on a 3-T Siemens Skyra Scanner (Siemens, Erlangen, Germany) equipped with a standard head coil. A total of 640 T2∗-weighted volumes of the whole brain [EPI-sequence; time to repeat (TR) 2000 ms, echo time (TE) 30 ms, flip angle 80°, and field of View (FOV) 192 mm, matrix 64^2^, 30 slices, slice thickness 3.5 mm, interslice gap 0.35 mm] near to standard bicommissural (AC-PC) orientation were collected. After the functional measurement a 3D high resolution T1-weighted volume for anatomical information (MPRAGE-sequence, 192 slices, FOV = 256 mm, isovoxel 1 mm, TR 2.5 s, TE 4.37 ms, flip angle 7°) was recorded. The subject’s head was fixed during the entire measurement to avoid head movements.

### fMRI Data Analysis

Analysis and visualization of the data were performed using Brain Voyager QX (Brain Innovation BV, Maastricht, Netherlands) software (Goebel et al., 2006). First, a correction for the temporal offset between the slices acquired in one scan was applied. For this purpose the data was interpolated. After this slice scan time correction a 3D motion correction was performed by realignment of the entire measured volume set to the first volume by means of trilinear interpolation. Thereafter, linear trends were removed and a high pass filter was applied resulting in filtering out signals occurring less than two cycles in the whole time course. Structural and functional data were spatially transformed into the Talairach standard space (Talairach and Tornoux, 1988) using a 12-parameter affine transformation. Functional EPI volumes were spatially smoothed with an 8 mm full-width half-maximum isotropic Gaussian kernel to accommodate residual anatomical differences across participants.

For the statistical model a design matrix including all conditions of interest was specified using a hemodynamic response function. This function was created by convolving the rectangle function with the model of Boynton et al. (1996) using Δ = 2.5, τ = 1.25 and *n* = 3. Thereafter, a multi-subject random effects (RFX) analysis of variance model (ANOVA) with stimulation (AV-congruent vs. AV-incongruent) as the first main within-subject factor and group (AS vs. HC) as the second main between-subject factor was used for identification of significant differences in hemodynamic responses. Main effects of both factors and their interaction were considered. The false discovery rate threshold of q(FDR) < 0.05 (Genovese et al., 2002) was chosen for identification of the activated voxels. The centers of mass of suprathreshold regions were localized using Talairach coordinates (Talairach and Tornoux, 1988) and the Talairach Daemon tool (Lancaster et al., 2000).

## Results

**FIGURE 1:**
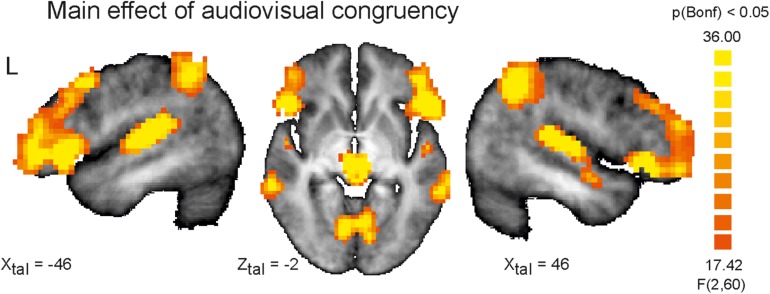
Depicted are sagittal views on the left (L) and right hemisphere and the horizontal view on the averaged brain of the measured population at Talairach coordinates *X* = 46, –46 and *Z* = –2. Color coded are voxels that survived conservative significance level of *p* < 0.05 corrected for multiple comparisons (Bonferroni, Bonf) for the main factor AV congruency. The use of this very conservative significance level was necessary for isolation of separated clusters due to the fact of widespread activation clusters covering almost the whole brain resulting from strong congruency effect and spatial smoothing at the same significance level but corrected for multiple comparisons by means of the false discovery rate.

Since each participant responded to almost all stimuli and the missing rate was nearly zero (ceiling effect) for each of the participants, all participants have attentively completed the experiments. Widespread differences of activation involving large parts of the brain were observed for the main within-subject factor congruency (AV congruent vs. incongruent) at the chosen significance level of q(FDR) < 0.05. Even after changing the significance threshold to *p* < 0.0001 (Bonferroni corrected for multiple comparisons) multiple significant clusters covering brain structures commonly involved in auditory and visual processing and attention functions were observed indicating the high impact of AV incongruency (Figure 1 and Table 2).

**TABLE 2:** Significant brain clusters identified for the main factor AV congruency.

**Regions**	**≈BA**	**Hemisphere**	**Talairach center of mass**			**Cluster size (mm^3^)**
			**x**	**y**	**z**	
*Incongruent* > *congruent*						
Inferior frontal gyrus	47	R	49	23	−3	621
Transverse temporal gyrus	41	R	48	−25	11	2592
Brainstem, Midbrain		R/L	−1	−20	0	2079
Medial frontal gyrus	8	L	−1	27	40	729
Transverse temporal gyrus	41	L	−44	−27	12	3996
Inferior frontal gyrus	47	L	−53	21	1	3051

**p*(Bonf) < 0.0001; BA Brodmann area; L left; R right.*

**FIGURE 2:**
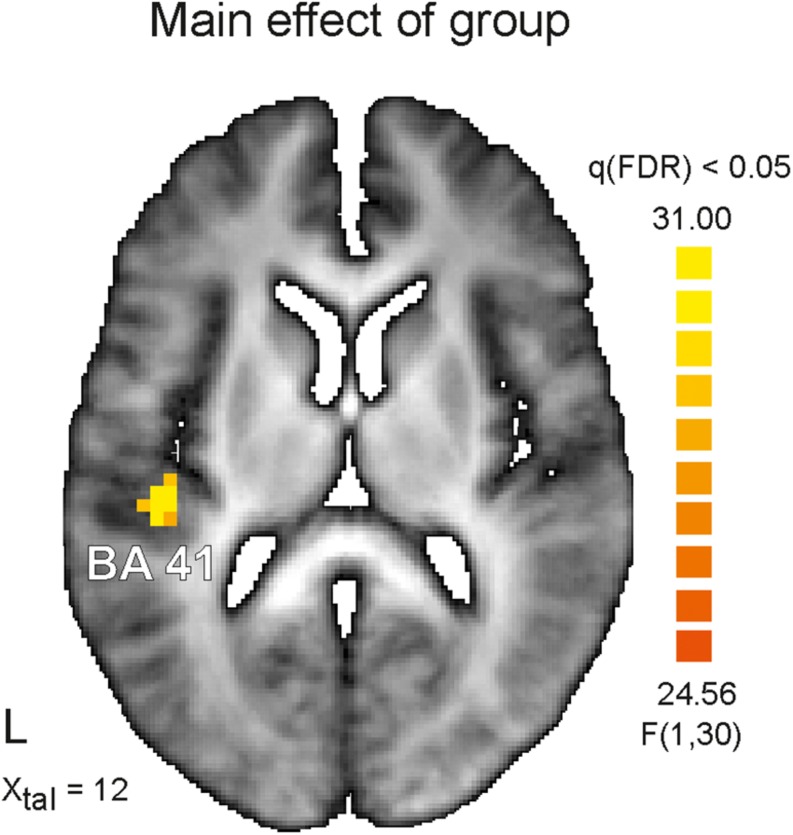
Depicted is horizontal view on the brain at the Talairach coordinate *X* = 12. On the left (L) hemisphere color coded cluster within the auditory cortex is shown. The identified cluster occupies Brodmann area 41 (BA41) and survived significance level of *q* < 0.05 corrected for multiple comparisons (false discovery rate, FDR) for the main factor Asperger’s syndrome. Healthy control subjects showed in this area stronger activation than Asperger’s subjects for both experimental conditions (audiovisual congruent and incongruent). Color coded is the *F*-value.

The main result of the study is a significant difference in activation for the main between-subjects factor (AS vs. HC). Figure 2 demonstrates the only one suprathreshold [q(FDR) < 0.05] cluster identified for this contrast. This left hemispheric cluster with an extent of 27 functional voxels occupies the transverse Temporal Gyrus and has its center of mass at Talairach coordinates (*x*, *y*, *z*) −42, −25, 12, i.e., Brodmann area 41. The analysis of mean betas extracted for this cluster and each group and condition revealed a stronger activation in this area for HC subjects (AV-congruent: 2.47 ± 0.64; AV-incongruent: 2.44 ± 0.73) in comparison to AS subjects (AV-congruent: 1.35 ± 0.75; AV-incongruent: 1.30 ± 0.81), *F*(1) = 21.05, *p* = 0.00, *n*^2^*_*p*_* = 0.41. The analysis of interaction of both main factors revealed no significant clusters at the chosen threshold level.

## Discussion

In this study we analyzed brain activity for AV congruent and incongruent speech stimuli in a group of adult AS subjects in comparison to typically developed healthy adult control subjects. We tested two competing hypotheses regarding multisensory processing deficits in ASD: Activity differences in brain areas typically associated with multisensory processing would indicate a primary deficit in multisensory processing occurring on later stages of the processing cascade. By contrast, group differences in unisensory brain areas would indicate rather low-level deficits which impact later multisensory processing.

The main finding of the study is a group difference in the left temporal cortex. As the mapping of the brain activity to specific brain locations within auditory cortex by mean of Talairach space leads to some systematic errors (Gaschler-Markefski et al., 2006) with a strong tendency for caudal displacement of BA 41 this activity cluster can by assigned to BA 41 and/or BA 42. Nevertheless both areas are known to support unisensory auditory processing stages at cortical level. While AS subjects show less activation in this cluster than healthy controls, this difference is independent of the AV congruency of speech stimuli. This finding therefore suggests a difference in unisensory auditory processing at a lower level of the processing cascade in AS.

As in several other experiments employing similar stimuli (Szycik et al., 2009a, b; Rüsseler et al., 2018), a very strong congruency effect was observed in the present study which involved superior temporal and frontal areas. In particular, the superior temporal sulcus (STS) typically displays increased activation for AV stimuli compared to unimodal presentation and also shows congruency effects (Calvert et al., 2000; Sekiyama et al., 2003; Wright et al., 2003; Brefczynski-Lewis et al., 2009; Szycik et al., 2009a, 2012; Lee and Noppeney, 2011; Nath and Beauchamp, 2011, 2012; Stevenson et al., 2011). Also, the inferior frontal cortex has been repeatedly shown to be involved in AV integration which has been interpreted as providing a motor plan for the production of the phoneme that the listener is about to hear (Skipper et al., 2007).

The fact that no group by congruency interaction effect is present in the current data set would suggest that AS participants have alterations in unisensory processing stages. Whereas the current results suggest low-level auditory processing differences in AS, it is puzzling that these changes do not propagate to subsequent multimodal processing stages either leading to compensatory overactivation or to decreased activation due to insufficient input. Moreover, the current experiment was designed against a background of multiple studies that have shown behavioral AV integration deficits in AS, but did not entail a behavioral readout assessing multisensory integration. Therefore, it is impossible to assess whether the decreased activity in left temporal cortex in AS has a behavioral correlate, or represents in contrast to typically developed subjects just more efficient neuronal processing of the auditory input at this stage. To address these points, further experiments are necessary linking activity differences in auditory and multisensory areas to behavioral effects (e.g., Rüsseler et al., 2015).

Previous studies of auditory perception in adults with AS have shown abnormalities in unimodal auditory processing even on low-level stages of the auditory stream (Marco et al., 2011). Thus, similarly to our results reduced activity in the left auditory cortex to non-speech auditory stimuli has been seen in adult patients with ASD (Hames et al., 2016). Additionally, weaker ERP signals of subjects with ASD measured by EEG in the temporal region and representing low-level cortical stages of the auditory system correlated to symptom severity in the autism spectrum (Brandwein et al., 2015).

On low-level stages of auditory processing the basic features of the incoming auditory information have to be extracted. These features may play a crucial role for successful integration of auditory and visual sensory input (e.g., Baumgart et al., 1999; de Heer et al., 2017). Indeed, previous studies have shown higher speech perception (about 3.5 dB) thresholds in AS subjects (Alcantara et al., 2004) which lead to a substantial decrease in speech recognition. Children with AS have been found to have considerable deficits in auditory stream segregation with reduced mismatch negativity amplitude when more than one sound stream is present (Lepisto et al., 2009) pointing to deficits in auditory processing. Similarly, neural responses to basic features of speech sounds within auditory cortex were found in other mismatch negativity experiment with AS children (Kujala et al., 2010).

To summarize, multisensory problems or problems in solving AV speech situations that subjects with AS often show may be related to deficits in ability to combine stimulus details or specific features into a coherent percept, as postulated by the “weak central coherence theory” (Happe and Frith, 2006). For AV speech stimuli this deficit seems to be related to alterations in unisensory auditory processing areas than in multisensory integration areas.

## Limitations

One problem with the current design is that the current experiment did not entail a behavioral task that assessed multisensory integration and additional unisensory stimulation. We made this design decision, as the current paradigm has been proven quite useful in assessing audiovisual integration during speech perception in healthy participants (Szycik et al., 2009a), patients with schizophrenia (Szycik et al., 2009b) or participants with dyslexia (Rüsseler et al., 2018). For this study we used a presentation mode with a lower stimulation frequency which resulted in a small amount of stimuli and a long overall experimental period. A low stimulation rate was designed to give the BOLD response sufficient time to relax after each audiovisual stimulus. However, as we only observed alterations in unimodal auditory areas, a behavioral read-out and unisensory condition would be very helpful for interpretation.

As multiple studies have demonstrated eye-movement abnormalities in AS (Baron-Cohen et al., 2001a; Klin et al., 2002; Pelphrey et al., 2002; Dalton et al., 2005; Neumann et al., 2006; Norbury et al., 2009), the question arises whether altered eye-movements in AS might contribute to AV speech integration. As most previous studies, we did not determine gaze-behavior and eye-fixations in the current experiment. Thus, we cannot speak to this question. One previous study, however, did not find eye-movement changes in AS in an experiment assessing AV integration in speech processing (Saalasti et al., 2012).

Another important limitation of this study concerns the diagnosis of AS. Since our participants were collected from a clinical population and the diagnostic procedures for this study started prior to widespread German adaptation of DSM-5, we used DSM-IV-TR diagnostic criteria for AS for all study participants. Therefore the results of this study refer strictly speaking only to a subpopulation of subjects with the diagnosis of ASD according to DSM-5.

## Data Availability Statement

All datasets generated for this study are included in the manuscript/Supplementary Files.

## Ethics Statement

All procedures performed in studies involving human participants were in accordance with the ethical standards of the institutional (Hannover Medical School) research committee and with the 1964 Helsinki declaration and its later amendments or comparable ethical standards. Informed consent was obtained from all individual participants included in the study.

## Author Contributions

F-AT participated in coordination of the study and the statistical analysis, performed the measurement, interpreted the data, and drafted the manuscript. LH and MR participated in coordination of the study and performed the measurement. MZ participated in coordination of the study and interpreted the data. CS participated in coordination of the study and the statistical analysis, performed the measurement, and helped to draft the manuscript. DW and MW participated in the statistical analysis, interpreted the data, and helped to draft the manuscript. TM participated in study design and the statistical analysis, interpreted the data, and drafted the manuscript. GS conceived of the study, participated in design and coordination of the study and the statistical analysis, interpreted the data, and drafted the manuscript. All authors read and approved the final manuscript.

## Conflict of Interest

The authors declare that the research was conducted in the absence of any commercial or financial relationships that could be construed as a potential conflict of interest.

## Supplementary Material

TABLE S1 Stimulus list.
